# Diabetes Mellitus and the Prostate: The Odd Pairing

**DOI:** 10.7759/cureus.90712

**Published:** 2025-08-22

**Authors:** Mohamad Fleifel, Andrew El Alam

**Affiliations:** 1 Endocrinology, Diabetes, and Metabolism, Bikhazi Medical Group, Beirut, LBN; 2 Endocrinology, Diabetes, and Metabolism, Ain Wazein Medical Village, Chouf, LBN; 3 Endocrinology and Metabolism, Dr. Sulaiman Al Habib Medical Center - Al Ghadeer, Riyadh, SAU; 4 Endocrinology, Diabetes, and Metabolism, Lebanese American University Medical Center, Beirut, LBN

**Keywords:** benign prostatic hyperplasia, diabetes, diabetes mellitus, prostate, prostate cancer

## Abstract

The prostate is a susceptible organ that could be impacted by metabolic diseases such as diabetes mellitus (DM), and more specifically type 2 DM (T2DM). Different literature sources suggest various associations between T2DM and prostatic diseases, most notably prostate cancer (PCa) and benign prostatic hyperplasia (BPH). This article serves as a comprehensive literature review on T2DM and anti-DM agents concerning PCa and BPH. Despite DM playing a role in contributing to the high risk of different malignancies and leading to worse mortality outcomes, multiple sources from the literature explain that DM might hold a protective effect against PCa. Several reasons could be discussed concerning such a lower risk, such as the dominant phenotype of T2DM patients, genomics, and T2DM’s impact on testosterone and prostatic vasculature. It also appears that the risk is associated with the time of T2DM diagnosis. Still, such a notion is not universally established, as some studies show that pre-existing DM in patients diagnosed with PCa increases mortality risk. DM might produce obstructive and irritative symptoms that mimic those of BPH. Such symptoms could be more challenging to respond to typical BPH therapy if glycemic control was not optimal. The dominant literature sources suggest that T2DM, obesity, and metabolic syndrome increase the risk of BPH. Metformin appears to be the most prominent anti-DM agent in terms of its positive effect on inhibiting PCa cell growth. Multiple trials are still ongoing to discover more of its anti-malignant roles. Conflicting reports are still present regarding multiple anti-DM agents and their true impact on PCa and BPH. It is vital to recognize that a relationship exists between prostatic diseases and T2DM. Close follow-up and screening are needed from both ends of the clinical evaluation.

## Introduction and background

The prostate is possibly one of the most sensitive organs when it comes to its response to environmental and metabolic stressors. Diabetes mellitus (DM) is regarded as one of the most renowned pathological metabolic states that the human body can exhibit. The relatively high prevalence and rising incidence of type 2 DM (T2DM) worldwide make it a topic of high interest when it comes to its association with other bodily diseases, including those of the prostate.

Prostate cancer (PCa) and benign prostatic hyperplasia (BPH) are probably the two most common and most studied prostatic diseases. Scholars suggest a conflicting association between T2DM and such diseases at times. For example, the pro-inflammatory state created by insulin resistance, which subsequently leads to the likely rise of T2DM, facilitates an environment that suits tumorigenesis [1,2]. This generates a scientifically arguable point that the metabolic-to-hypermetabolic state of T2DM fits the rise of PCa. However, subjectively, an argument could be made regarding a lower risk of PCa in patients with advanced/chronic T2DM and those who also suffer from obesity on top of their diabetes, regarding poor vascularity and low testosterone level, respectively. When it comes to BPH, based on clinical experience, the lines also seem to be blurred for BPH symptoms in patients with T2DM, as urologic symptoms can exist in such patients even in the absence of a clear-cut BPH diagnosis. In addition, as we mentioned before, given how sensitive the prostate is, it is likely that anti-DM agents impact the prostatic function and cellular growth as well. Therefore, we aim to go over multiple sources in the literature that have tried to establish a relationship between diabetes and the prostate while also providing our subjective scientific opinions on the matter. The article aims to provide a comprehensive literature review on T2DM and anti-DM agents concerning PCa and BPH.

## Review

Prostate cancer and diabetes mellitus: the paradox

Most of the studies have been done on the dynamics between prostate diseases, including cancer, and T2DM instead of the autoimmune type 1 DM (T1DM). T2DM has been reported to be positively associated with a higher risk of certain malignancies, including pancreatic, hepatobiliary, renal, bladder, colorectal, oropharyngeal, breast, endometrial, ovarian, leukemia, glioma, and melanoma [1-5]. A 2015 meta-analysis concluded that T2DM was associated with around a 10% increase in total cancer risk [6]. Some studies have even suggested that DM plays a role in premature cancer deaths compared to those who do not suffer from the metabolic disease [7]. The association between DM and total cancers could be due to shared modifiable and non-modifiable risk factors like aging, ethnicity, gender, obesity, unhealthy diet, poor physical activity, and smoking [2]. Certainly, the individual cancer link with DM likely differs on a pathophysiologic level. For example, pancreatic cancer animal models suggest a prominence of hyperinsulinemia and significant impairment of insulin secretion; therefore, both insulin sensitivity and β-cell function are impaired [8-10]. A vicious cycle can exist between hyperinsulinemia and inflammation, as the former favors the release of reactive oxygen species (ROS). This encourages the persistence of a chronic inflammatory state, which is characterized by high levels of oxidative stress. That creates a hypermetabolic milieu of unremitting inflammation exhibited through pro-inflammatory cytokines and abnormal adipokine production, which can promote tumorigenesis, angiogenesis, and metastasis while also impairing the immune system’s lines of defense in the macrophages and natural killer cells. Prominent ROS further incapacitates the cardiometabolic profile of the individual and promotes further hyperinsulinemia [11,12]. Hyperglycemia, which is the hallmark of DM, favors DNA damage, which is one of the initial stages of tumorigenesis of the lung, colorectal, hepatic, gastric, and pancreatic cancers [13]. This is explained as cancer cells rely on a less efficient glycolytic pathway for growth and proliferation; thus, they need more glucose uptake to generate sufficient energy to meet their proliferation requirements. This is sometimes referred to as “the Warburg effect” [14,15].

Interestingly, despite all the described proposed pathophysiology, multiple studies from the literature suggest that DM patients have a lower PCa incidence, unlike the rest of the mentioned malignancies. The Health Professionals Follow-Up Study (HPFS) from 1986 to 1994 noted 1,369 new cases of non-stage A1 PCa in 47,781 men. Those with T2DM did not exhibit any PCa risk reduction in the first five years after diagnosis; however, the risk was lower in the next five years and the lowest post 10 years of diagnosis (RR = 0.54, CI = 0.37-0.78, p = 0.004) [16]. A follow-up study to the HPFS by Lutz et al. reported that PCa patients tend to display elevated fasting serum blood glucose (SBG) levels and insulin resistance, without any changes in insulin secretion. This was based on an age and body mass index (BMI)-matched comparison between 103 newly diagnosed and untreated PCa patients and 107 healthy controls. The authors excluded patients with overt DM to avoid any possible interference by DM medication. The same study did not find any significance when comparing the two samples’ c-peptide. The authors also concluded that patients who had DM and higher BMI were less likely to develop PCa with time [17]. Therefore, both studies suggest that, with temporal progression, the risk of PCa diagnosis decreases when DM is initially revealed.

Still, the relationship between PCa and DM remains paradoxical in clinical terms. Elaborating from clinical experience, many patients with various cardiometabolic disorders develop PCa regardless of the time since the diagnosis of the metabolic disease, which includes DM. General practitioners, urologists, or endocrinologists will probably not reassure a DM patient concerning a lower risk of PCa, regardless of what the literature predominantly suggests. From a pathophysiological standpoint, explanations for DM having a statistically “protective” role against the rise of PCa could be that those patients with T2DM, especially chronic disease (≥10 years duration), develop lower levels of testosterone than those with recent T2DM onset or no DM. Testosterone is linked to an increased risk of PCa; thus, a decrease in its level, as a consequence of the dynamic relationship between insulin resistance and testosterone level, could make T2DM patients less likely to have PCa [18,19]. In addition, given that many T2DM patients also suffer from overweight or obesity, which likely results in lower serum testosterone, then that would reflect lower PCa incidence as per Lutz et al.'s findings. Another reason could be the vascular complications that DM patients present with over time. It is postulated that with long-standing DM, prostatic microvascular compromise is probable. Therefore, a poor vascular supply would render any possible tumor growth unlikely [20]. This hypothesis does exhibit some valid arguments, as patients with long-standing DM and/or those with poorly controlled DM tend to have higher macrovascular and microvascular complications. Thus, a poor vasculature of the prostate in DM patients can lead to an ischemic-like event of the prostate, which prevents any probable tumorigenesis [21]. Therefore, this agrees with the data showing that the more chronic DM is, the less likely that such patients will develop PCa.

Genomics suggests that DM individuals with the HNF1B gene variants (i.e., monogenic diabetes type 5/maturity-onset diabetes of the young type 5 (MODY5)) are more protected from PCa, so this would subsequently hinder any tumor-related growth factors [22]. Regardless of how interesting these gene variants are, more studies on a large population scale need to be done to reinforce such an association. HNF1B variants tend to predispose individuals to DM (in particular MODY5 and not T2DM), which is a serious metabolic disease that, if left uncontrolled, could lead to drastic cardiovascular outcomes. Therefore, reporting on gene variations as a possible prognostic factor for survival from PCa does not hold concerning severe cardiovascular morbidity and probable mortality outcomes. Future studies on specific gene variants linked to T2DM that also hold significant prognostic value for PCa are crucial for future research. With DM, the actual prostate size and circulating prostate-specific antigen (PSA) are affected [22,23]. A study from Iraq showed that the mean total PSA was lower among T2DM versus non-T2DM males (1.97 ± 1.05 ng/ml versus 2.60 ± 1.22 ng/ml, respectively, p = 0.001) [24]. However, such DM-to-PSA correlation does not seem to be unanimous. For example, in a Moroccan cross-sectional study of 470 DM and 869 non-DM males, PSA was only lower in DM subjects aged 50-59 years as compared to non-DM individuals. PSA levels increased with age in DM and non-DM subjects, and serum testosterone did not play a significantly different role between the two groups [25]. A large cross-sectional study from Japan noted that serum PSA levels were significantly reduced in men with T2DM on anti-diabetes drugs, higher glycated hemoglobin A1c (HbA1c), higher fasting SBG levels, obesity, and elevated alanine transaminase [26]. Such findings and associations should be carefully interpreted and individualized based on the clinical setting and the patient. Men with BMI, which is a predominant profile in T2DM and individuals with metabolic syndrome, likely exhibit hemodilution from increased plasma volume and lower androgen levels, which might facilitate a decrease in prostate size and lower the PSA [27,28].

However, such a “protective effect” of T2DM does not seem to be universally reported, as meta-analyses show that multiple Asian countries, notably the Far East, have an increased risk of PCa among T2DM men [29,30]. Therefore, despite the reported lower incidence of PCa with T2DM progression, one must never disregard the comorbidities that uncontrolled T2DM delivers with time. Based on several studies, pre-existing DM in patients diagnosed with PCa increases mortality risk. According to a study from Sweden, Liu et al. reported that pre-existing T2DM exhibited a 32% increased mortality risk among PCa patients [31]. A 17-cohort meta-analysis by Lee et al. documented that pre-existing DM (whether T2DM alone or both T1DM and T2DM) had a 29% increase in PCa-specific mortality and a 37% increase in all-cause mortality. However, T2DM’s association with mortality from PCa was statistically insignificant compared to the significant association seen for total DM [32]. Both Cai et al. and Snyder et al. reported that pre-existing DM was associated with increased risk of PCa mortality [33,34].

The arguments made for the lower incidence of PCa concerning the time of DM diagnosis are very plausible, especially concerning lower androgen levels and prostate microvasculature. The vicious cycle, which was described earlier, between low testosterone and hyperinsulinemia with or without obesity, is probably the most valid of the two. This is further reinforced as androgen deprivation therapy (ADT) is regarded as one of the major treatments for PCa; however, this therapy has been found to possibly promote hyperglycemia and hyperinsulinemia, thus proving a likely dynamic relationship between the two hormonal entities of insulin and androgens [35]. A meta-analysis by Wang et al. showed that >6 months of ADT (GnRH alone, GnRH + antiandrogen + orchiectomy) increased the incidence of T2DM by around 39% [36]. Prostate vasculature is regarded as very fragile, which is a trait that increases with aging. Examining how DM affects penile vessels and nerves and subsequently contributes to erectile dysfunction, it is probable that as DM progresses, or if it is left uncontrolled, the disease would eventually impact the fragile prostate vessels and nerves in a similar fashion to penile vascular impotence. A compromised cellular structure of the prostate will likely limit any significant tumorous growth. PSA’s usage as a marker should be carefully interpreted due to its relatively easy fluctuations in response to stressors. The role of anti-DM medications will be discussed in a separate section of the article, as the literature suggests a potential association between their use and a lower PCa incidence. One important factor that should be addressed is the possibility of PCa being underdiagnosed in DM patients. Low-grade or early-stage PCa with slow progression might be disregarded by the physician or patient if not proven through core needle biopsy and imaging. Like with BPH, if symptoms exist, they might be attributed to age or the collective effects of several diseases that the patient has by that time. The seriousness of DM and its cardiovascular, cerebrovascular, nephrotic, neuropathic, and retinopathic complications might dominate the concerns of physicians and/or patients. Still, as detailed before with DM’s role in total cancers, it cannot be taken as a straightforward rule that DM, hyperinsulinemia, obesity, and hyperglycemia are “positive” contributors that eliminate the risk of PCa.

Benign prostatic hyperplasia and diabetes mellitus: the camouflage

Predominant literature sources highlight that DM significantly increases the risk of BPH. Hammarsten et al. showed that patients with T2DM and lower urinary tract symptoms (LUTS) exhibited larger prostate volumes than patients with LUTS but without DM. This was also seen among patients with obesity, hyperinsulinemia, low high-density lipoprotein (HDL)-cholesterol levels, and those on antihypertensive medications [37]. Still, it is difficult to differentiate between LUTS secondary to DM versus those secondary to BPH. The concept of “diabetic cystopathy” has been previously described in the sense of damaged visceral afferent fibers in the bladder wall leading to compromised bladder sensation and contraction [38]. This produces obstructive and irritative symptoms, something that is also common in BPH patients without DM. LUTS in DM and DM + BPH patients may be more difficult to combat therapeutically, given the stubborn nature of DM complications. Such patients’ symptoms may be less affected by traditional BPH medical treatments, such as 5-alpha-reductase inhibitors (5-ARI) and/or alpha-blockers (AB). Some studies have shown that obstructive and irritative symptoms are more common among DM patients [39,40]. It is interesting to note that a study by Johnstone et al. reported a higher risk for new-onset T2DM among patients on BPH medical treatment when compared to those men without the treatment. The hazard ratios (HR) for the risk of T2DM among the medical therapies were 1.30, 95% confidence interval (CI) 1.25-1.35 for combination therapy of AB + 5-ARI, 1.17, 95% CI 1.13-1.22 for AB, and 1.25, 95% CI 1.17-1.34 for 5-ARI [41]. Still, other studies do not show such a relationship between DM and AB or 5-ARI, with some even suggesting a decrease in DM risk in BPH patients, which argues against the results from Johnstone et al.'s study [42-44].

Overall, one cannot deny the impact of hyperinsulinemia, obesity, and metabolic syndrome, all being predecessors to T2DM, as culprits in the risk of BPH. A study examining the correlation between insulin and lipid profile levels in non-DM BPH patients and prostate size found that insulin levels were significantly associated with prostate size. In addition, serum insulin, homeostatic model assessment (HOMA), total cholesterol, and low-density lipoprotein (LDL) cholesterol were higher in BPH cases, and HDL cholesterol was also lower in such patients as compared to controls [45]. In a study that evaluated the annual prostate growth rate in BPH patients, the authors highlighted the cases according to the presence or absence of metabolic syndrome. It was reported that patients with BPH-metabolic syndrome had a higher PSA when compared to those with BPH non-metabolic syndrome. Moreover, the median annual total prostate growth rate and median annual transitional zone growth rate were also higher in the BPH-metabolic syndrome group [46].

Abnormalities in glycemic homeostasis likely affect the prostatic cells’ proliferation rate, thus favoring hyperplasia. It is possible that in states of hyperinsulinemia, insulin would overpromote further prostatic tissue growth, considering that insulin is a growth-stimulating hormone. This is reinforced by the fact that insulin-like growth factor (IGF) is known to exist in prostatic tissue, and any pathologic anomalies in the IGF system are noted to be present among the prostatic stroma cells in BPH patients [47,48]. It is postulated that androgens might regulate the IGF activity since experiments have shown that in the absence of androgens, IGF-1 receptors tend to decrease along with the IGF-1 mRNA and the IGF binding proteins [49,50].

From our point of view, in clinical settings, T2DM and other metabolic diseases might contribute to the rise or exacerbation of BPH or BPH-like symptoms. Therefore, in addition to the more common literature-based nephrological and genitourological complications like diabetic neuropathy, neurogenic bladder, and erectile dysfunction, BPH symptoms and complaints should always be evaluated for. In addition, it might be a good choice to look for metabolic diseases in patients with BPH carefully.

**Figure 1:**
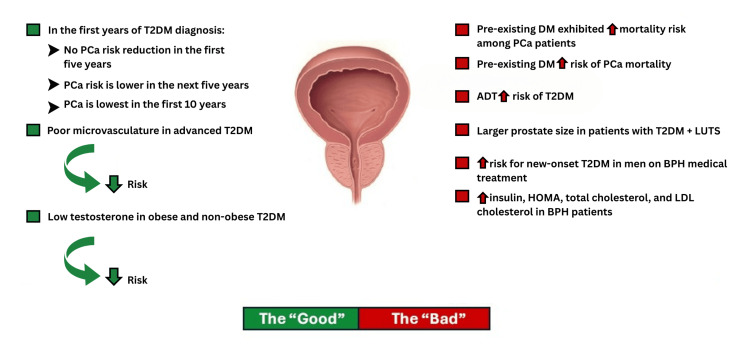
The "good" and "bad" of the diabetes and prostate relation The figure summarizes the points discussed in the first two subheadings of the review. Downward green arrow: risk decreased, upward red arrow: risk increased, DM: diabetes mellitus, T2DM: type 2 diabetes mellitus, PCa: prostate cancer, BPH: benign prostatic hyperplasia, ADT: androgen deprivation therapy, LUTS: lower urinary tract symptoms, HOMA: homeostasis model assessment, LDL: low-density lipoprotein Image Credit: Dr. Mohamad Fleifel. Prostate model image edited. Original file can be found at https://upload.wikimedia.org/wikipedia/commons/7/73/Normal-vs-enlarged-prostate.jpg. WikiMedia Commons; licensed under the Creative Commons CC BY-SA 3.0 Attribution-Share Alike 3.0 Unported license (https://creativecommons.org/licenses/by-sa/3.0/deed.en)

The points discussed in this article were included as bullet points as part of the "good and bad" of the dynamic relationship between DM and PCA/BPH (Figure 1). An essential note is that the understanding of such a relation is still infantile, in our opinion, as clear-cut findings are yet to determine if DM "increases" or "decreases" the risk of PCa or BPH, or even definitely exhibits higher overall mortality or death from genitourological complications related to the prostate. This article generally lays out some of the data that was found in the literature review.

Role of anti-diabetic medications: the good, the bad, and the unknown

The collateral effects of certain medications on other bodily organs, whether positive or negative, have always been topics of research over the years. Investigating anti-DM agents’ impact on the prostate and prostate diseases is of valid importance.

Metformin, currently the sole member of the biguanide family, promotes anabolic metabolism over the catabolic one, thus playing a role in the inhibition of cell proliferation. Therefore, this favors anti-inflammatory and anti-cancer effects [51]. It is thought that metformin’s anti-proliferative effects in PCa cells are linked to AMP-activated protein kinase (AMPK)-independent inhibition of the cell-cycle regulator cyclin D1 in the G0/G1 phase; therefore, an energy source deprivation would ultimately lead to cellular apoptosis [52]. Such an important aspect of metformin on cancer cells has been observed among other malignancies through the same or similar antiapoptotic mechanisms. Such malignancies are esophageal, lung, and breast cancers [53-55]. In addition, it has been shown that metformin’s antiapoptotic effects are possibly more effective when used with other drugs, notably anti-cancer agents that prevent glycolysis [56,57]. In a study of 211,648 BPH-naive patients who were diagnosed with BPH in 2009 and had a follow-up occurrence of prostatectomy until 2017, defined as BPH progression, the T2DM with high-dose metformin patient group had a significantly lower risk of future prostatectomy. Therefore, it was concluded that metformin could halt the progression of BPH [58]. However, studies remain inconsistent regarding metformin’s definitive role in prostate diseases. A retrospective study by Liu et al. demonstrated that metformin users in PCa patients presenting for radiation therapy may exhibit lower levels of PSA.

However, the treatment outcomes were not impacted [59]. This trend in PSA lowering among metformin users does not always seem to be of significance [60,61]. Promising results exist from animal studies, as metformin administration to mice with xenografts of human PCa cells contributed to the reduction of tumor growth by 35% to 50%, which was attributed to the fall in cyclin D1 activity [52]. Furthermore, Akinyeke et al. showed that metformin inhibits PCa through reducing the oncogene c-myc [62]. Metformin has also been shown to help in ceasing cellular proliferation in BPH in dose- and time-dependent manners [63]. El-Arabey et al. dubbed metformin the “superdrug” based on its multiple vibrant pharmacokinetics and its ability to enter and affect multiple body tissues [64]. We agree with such a statement, as multiple clinical trials are currently in motion regarding the effects of metformin on cancer cells as an adjuvant therapy, including those of the prostate.

In a systematic review and meta-analysis by Cui et al., 3,094,152 DM patients were analyzed, and results showed no significant association between PCa and metformin, sulfonylureas (SUs), thiazolidinediones (TZDs), dipeptidyl peptidase-4 inhibitors (DPP4-is), or insulin. Side analysis of the randomized controlled trials exhibited a reduction in PCa risk with TZD or glucagon-like peptide-1 receptor agonist (GLP-1RA) usage. No significant association was found for sodium glucose cotransporter-2 inhibitors (SGLT-2is) [65]. In the Liraglutide Effect and Action in Diabetes: Evaluation of Cardiovascular Outcome Results (LEADER) trial, it was shown that PCa was present in DM patients at a lower proportion among liraglutide users, as compared to the placebo group [66]. It has been demonstrated that liraglutide and exenatide inhibit cell proliferation and increase apoptosis in PCa cells through an increase in the ratio of pro-apoptotic Bax/anti-apoptotic Bcl-2 and promote the activation of the p38 mitogen-activated protein kinase-dependent cell death pathway [67]. Both exenatide and metformin have an additive effect on the reduction of the PCa tumor size and the inhibition of cell growth [68]. According to our literature review, there are no studies detailing any significant relationship between semaglutide or tirzepatide and PCa or BPH. Considering that semaglutide is a GLP-1RA and tirzepatide is a dual GLP-1RA/glucose-dependent insulinotropic polypeptide, their effects might mirror those of liraglutide and exenatide when it comes to the prostatic diseases. A decrease in body weight and fat could help promote an anti-inflammatory state characterized by low ROS. This would subsequently decrease the risk for PCa and BPH to some extent. However, large sample size studies are likely needed to prove that.

A study from Stockholm, Sweden, revealed that serum PSA levels did not vary between men who were on metformin, SUs, or insulin when compared to those unexposed to any of the anti-DM medications. Still, the probability of prostate biopsy after elevated PSA levels was lower among men receiving metformin and insulin [69]. Such results could be based on the technical side of patient follow-ups, which could manifest as lower compliance to actual clinic visits after an anti-DM agent is prescribed for them, or the lack of careful prostate follow-up from general practitioners and/or endocrinologists in the absence of clear-cut prostate-related symptoms. Another study found no protective effects of metformin, insulin, aspirin, or statins against PCa risk [70]. In a Hong Kong-based study comparing PCa risks between SGLT-2is and DPP4-is in T2DM patients, the risk was lower among users of SGLT-2is. It appeared that SGLT-2is reduced PCa risks significantly among elderly patients (age >65), those with second and third HbA1c quartiles, concurrent metformin users, and concurrent SU users. A higher PCa risk was seen among those who used SGLT-2is but not SUs [71]. SGLT-2is may work on PCa cells similarly to their effects on breast cancer cells. A study showed that dapagliflozin and canagliflozin exhibited a potent anti-proliferative role among breast cancer cells through AMPK-mediated cell cycle arrest and apoptosis [72]. Outside of metformin to some extent, there seems to be a never-ending conflicting loop when it comes to the influence that anti-DM agents might have on prostate diseases. As is the case with the prior two subheadings, we believe that the field of antidiabetes agents and significant prostatic effects has not been dwelt upon in a significant and comprehensive manner (Table 1).

**Table 1:** The "good" and the "bad + unknown" of the literature findings of anti-diabetes medications with respect to PCa and BPH The table summarizes the findings that were discussed in the text. Notice how the "bad + unknown" are predominantly not negative effects, but the limited findings in the literature concerning anti-diabetes medical therapy and prostatic consequences. DM: diabetes mellitus, PCa: prostate cancer, BPH: benign prostatic hyperplasia, PSA: prostate-specific antigen, ROS: reactive oxygen species, TZDs: thiazolidinediones, GLP-1RAs: glucagon-like peptide-1 receptor agonists, SGLT-2is: sodium-glucose transport protein 2 inhibitors

Antidiabetes medication	The “good”	The “bad” + "uknown"
Metformin	Anti-inflammatory and anti-cancer effects, antiapoptotic effects are more effective when used with anti-cancer agents, decrease tumor size in PCa, stop BPH progression, and lower levels of PSA	Radiation therapy outcomes in PCa were not affected, some studies still show no significance
TZDs	Reduction in PCa risk (side analysis)	Most studies still show no significance
GLP-1RAs	Inhibit cell proliferation and increase apoptosis in PCa cells, decrease tumor size in PCa (exenatide), decrease PCa proportions in men with DM (liraglutide), losing weight promotes an anti-inflammatory state with low ROS, which might lower the risk for BPH and PCa	Some studies still show no significance
SGLT-2is	Decrease PCa risk	Some studies still show no significance

## Conclusions

The proper handling of prostatic diseases among patients with T2DM is essential, whether such a metabolic disease favors their progression or not. It is evident that a relationship exists between T2DM and its elements on one end and PCa and BPH on the other. Multiple molecular pathways have been suggested to explain such a dynamic relationship; however, the importance lies in realizing that patients need screening for prostatic diseases in the setting of T2DM and vice versa.
